# Pathogenic tau decreases nuclear tension in cultured neurons

**DOI:** 10.3389/fragi.2023.1058968

**Published:** 2023-01-23

**Authors:** Claira Sohn, Jiacheng Ma, William J. Ray, Bess Frost

**Affiliations:** ^1^ Department of Cell Systems and Anatomy, Barshop Institute for Longevity and Aging Studies, Glenn Biggs Institute for Alzheimer’s and Neurodegenerative Diseases, University of Texas Health San Antonio, San Antonio, TX, United States; ^2^ The Neurodegeneration Consortium, Therapeutics Discovery Division, The University of Texas MD Anderson Cancer Center, Houston, TX, United States

**Keywords:** tauopathy, FRET, nuclear tension, Alzheimer’s disease, nucleus, mechanobiology

## Abstract

Neurodegenerative tauopathies, including Alzheimer’s disease, are pathologically defined by the presence of aggregated forms of tau protein in brains of affected individuals. Previous studies report that the negative effects of pathogenic tau on the actin cytoskeleton and microtubules cause a toxic destabilization of the lamin nucleoskeleton and formation of nuclear invaginations and blebs. Based on the known function of the nucleus as a mechanosensor, as well as the high incidence of nuclear pleomorphism in human Alzheimer’s disease and related tauopathies, we investigated the effects of pathogenic tau on nuclear tension. We first find that tau-dependent nuclear envelope invagination and relocalization of LInker of Nucleoskeleton and Cytoskeleton (LINC) complex components are conserved in a newly-developed neuroblastoma cell line that features doxycycline-inducible expression of a tau mutant associated with autosomal dominant frontotemporal dementia. We next determine that a Förster resonance energy transfer (FRET)-based sensor of nuclear tension responds to cytoskeletal stabilization and destabilization when expressed in neuroblastoma cells. Using this nuclear tension sensor, we find that induced expression of pathogenic tau is sufficient to decrease nuclear tension. This work provides the initial proof-of-concept evidence that pathogenic forms of tau alter nuclear tension, paving the way for the future study of altered nuclear mechanosensing in the context of tau-mediated neurodegenerative disorders.

## 1 Introduction

The human body undergoes constant mechanical stress. With each footstep or beat of the heart, cells stretch and squish to maintain organ integrity and function. Despite being an immobile organ that is protected by the skull, the brain is also subjected to mechanical stress. An extensive brain vasculature promotes the constant flow of blood throughout the brain and maintains the blood brain barrier. Brain vasculature directly interacts with cells of the brain, causing each cell to experience a different level of mechanical stress. In rats, for example, somatosensory stimulation increases the diameter of single pial arterioles by 30% (Ngai and Winn, 1996). The presence of stress-sensitive ion channels such as TRAAK (Maingret et al., 1999) and TREK-1 (Hervieu et al., 2001) in the plasma membrane of neurons further suggests that these cells are poised to respond to mechanical cues that result from changes in the diameter of the vasculature that occur due to differences in blood flow. Indeed, studies in cultured neurons indicate that mechanical stimulation induces a neuronal calcium response (Gaub et al., 2020).

**FIGURE 1:**
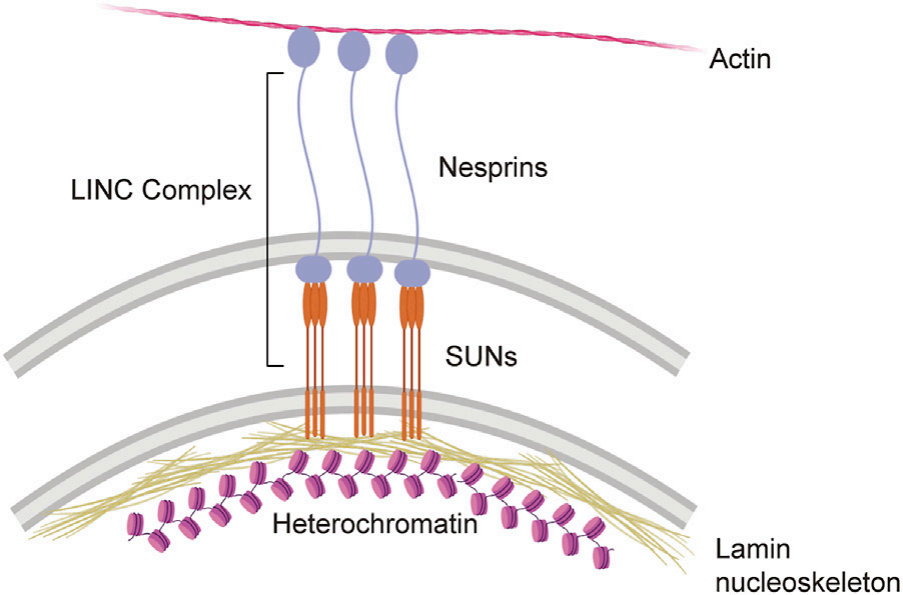
The LINC complex and its direct interactions with the cyto- and nucleoskeleton.

While it is clear that cells experience mechanical stress and that this mechanical stress can change under physiological and pathological conditions, relatively little is known in regard to the mechanosensing abilities of cells within the brain (Kirby and Lammerding, 2018). Among cells in the periphery, a significant body of work in mechanobiology has identified the cellular nucleus as a critical detector and responder to mechanical stress (Kirby and Lammerding, 2018; Maurer and Lammerding, 2019; Hamouda et al., 2020; Janota et al., 2020). Recent studies in human epithelial progenitor cells suggest that the nucleus undergoes mechanical softening to protect against mechanical stress-induced damage (Nava et al., 2020). Mechanical cues are communicated from the cytosol to the nucleus (and vice versa) *via* the nuclear envelope-spanning LINC complex (Figure 1). Within the LINC complex, lamin-bound SUN proteins form a trimer that extends from lamin intermediate filaments in the nucleus into the perinuclear space to directly bind to nesprin proteins. Nesprins extend from the perinuclear space into the cytoplasm where they can bind directly to actin filaments, interact with microtubule motors, and bind indirectly to intermediate filaments (Janota et al., 2020). While the mechanosensing properties of the LINC complex have not been investigated in neurons, studies in primary human mesenchymal stem cells report that strain on the LINC complex directly influences cell shape, chromatin compaction, post-translational modifications, and the proteome (Gilbert et al., 2019).

An accumulating body of work indicates that the lamin nucleoskeleton undergoes destabilization and invagination/blebbing in cells harboring pathogenic forms of tau (Frost et al., 2016; Montalbano et al., 2019; Paonessa et al., 2019; Jiang and Wolozin, 2021). As wild-type and mutant forms of tau protein can assemble into soluble toxic multimeric species, large neurofibrillary tangles and inclusions, and can drive neurodegeneration in *Drosophila* by undergoing disease-associated posttranslational modification and conformational changes in the absence of detectable multimerization (Wittmann et al., 2001; Steinhilb et al., 2007), we define “pathogenic” forms of tau as those carrying phosphoepitopes that are associated with human disease for the purposes of the current study. Alongside an overall reduction of lamin protein in brains of *Drosophila* models of tauopathy and in human brains affected by Alzheimer’s disease, neuronal nuclei feature a widespread decondensation of heterochromatin and extensive nuclear invagination and blebbing. In *Drosophila,* genetic manipulation of actin polymerization and LINC complex components mediates tau-induced neurodegeneration and nuclear architectures changes, demonstrating a causal link between the effects of tau on the actin cytoskeleton, the LINC complex, the lamin nucleoskeleton and neurodegeneration (Frost et al., 2016). Recent studies in cultured cells and primary mouse tissue suggest that disruption of nuclear envelope integrity is a possible initiating event in tauopathies (Prissette et al., 2022). While there is thus a clear breakdown of the nucleoskeleton and morphological changes in neuronal nuclei in the context of tauopathy, no study to date has investigated the mechanical properties of nuclei within cells harboring pathogenic forms of tau.

A foundational advance in mechanobiology research involved development of the FRET-based tension sensor module “TSmod” (Grashoff et al., 2010). TSmod features an elastic peptide flanked by mTFP1 and Venus fluorescent proteins. Force input on TSmod causes elongation of the elastic peptide, which reduces the overall FRET signal by reducing the energy transferred from the mTFP1 donor to the Venus acceptor. The TSmod biosensor has been inserted into a variety of proteins that reside at intercellular and cell-matrix adhesion sites in order to quantify overall cellular tension (Grashoff et al., 2010; Borghi et al., 2012; Conway et al., 2013; Cai et al., 2014; Kuriyama et al., 2014; Austen et al., 2015). In the current study, we utilize a version of TSmod that is inserted into a functional Nesprin-2G construct that allows quantification of mechanical forces between the actin cytoskeleton and the nuclear envelope (Arsenovic and Conway, 2018). In recent years, this nesprin tension sensor (nesprin-TS) has been utilized to measure mechanical forces on the LINC complex in fibroblasts, embryonic stem cells, myofibroblasts, and epithelial cells (Arsenovic and Conway, 2018; Hu et al., 2019; Walker et al., 2021; Pothapragada et al., 2022).

Here we combine an inducible model of tauopathy with the nesprin tension sensor to discover that induced expression of pathogenic tau causes an overall reduction in nuclear tension in cultured neuroblastoma cells. These results highlight the mechanosensing properties of the nucleus in neurons and lay the groundwork for future investigation into the effects of pathogenic forms of tau on nuclear mechanotransduction.

## 2 Materials and methods

### 2.1 cDNA constructs and viruses

*MAPT* cDNA (NM_005910.6) with a single-mutation (1216C>T) and *GFP* cDNA were synthesized and inserted into the doxycycline-inducible lentiviral vector PLIX_403 (Addgene #41395). Lentiviral particles were generated by the MD Anderson Functional Genomics Core Facility.

### 2.2 Cell culture and iTau stable cell line establishment

Human neuroblastoma cells (BE(2)-C; ATCC #CRL-2268) were cultured in a 1:1 mixture of Eagle’s Minimum Essential Medium (EMEM) and F12 medium supplemented with 10% tetracycline-free FBS and 1% penicillin-streptomycin. Stable cell pools of BE(2)-C_MAPT-R406W and BE(2)-C_GFP were generated by transducing BE(2)-C cells with PLIX_403_MAPT-R406W or PLIX_403_GFP lentiviral particles, followed by selection with 2 μg/mL puromycin. Expression of MAPT-R406W or GFP was induced with 1 μg/mL doxycycline hyclate dissolved in DMSO.

Cells were plated in antibiotic-free OptiMEM (low serum media) (ThermoFisher, #31985062) 24 h prior to transfection at a density allowing for ∼80%–85% confluency the following day. Cells were transfected with nesprin-TS or nesprin-HL (Addgene #68127 and #68128, respectively) using Lipofectamine 2000 (ThermoFisher, #11668030). 1 µg of DNA and 2.5 µL of Lipofectamine were diluted in 96.5 µL of OptiMEM for each well of a 12-well plate. The cocktail was incubated for 25 min at room temperature before adding to the cells. Cells were incubated with DNA/Lipofectamine in OptiMEM for 6 h prior to media replacement. Cells were collected 48 h after transfection for their respective experiments.

For doxycycline induction of transgene expression, cells were plated in antibiotic-free low serum media (EMEM supplemented with 2.5% tetracycline-free FBS) 24 h prior at a density allowing for ∼85%–90% confluency the following day. Cells were incubated with 1 μg/mL of doxycycline or vehicle (DMSO) for approximately 24 h before collection.

### 2.3 Western blotting

Cells were harvested at ∼90% confluency using RIPA with 1x protease inhibitor (Halt Protease, ThermoFisher) and then incubated at 4°C for 30 min with gentle rocking. Cell lysates were centrifuged for 20 min at 4°C at 12,000 rpm. A Bradford assay was performed on cellular supernatants to quantify protein concentration (BCA Protein Assay Kit, Pierce) prior to Western blotting. Protein lysates were boiled in 2x Laemmli buffer for 5 min, centrifuged for 1 minute at 12,000 rpm, then loaded onto a 4%–20% SDS–PAGE gel. 20 μg of protein was loaded per well. Equal loading was assessed by Ponceau S staining of nitrocellulose membranes after transfer. Membranes were blocked in PBS containing 0.05% Tween and 2% milk, and then incubated with primary antibodies overnight at 4°C. After washing, membranes were incubated with HRP-conjugated secondary antibodies for 2 h at room temperature. Blots were developed with an enhanced chemiluminescent substrate. Densitometry was performed using ImageJ.

### 2.4 Immunofluorescence

Cells were plated in 12-well plates on 20 mm coverslips prior to staining. Cells were fixed in 100% ice-cold molecular grade methanol at room temperature for 5 min, then washed three times with 0.01% Tween-20 in PBS for 5 min per wash. After washing, cells were permeabilized in 0.01% PBS Triton X-100 with 1% Bovine Serum Albumin (BSA) for 15 min at room temperature, washed three times with 0.01% Tween-20 in PBS, then blocked for 30 min in PBS with 1% BSA and 0.01% Tween-20. Primary antibodies were diluted in 1% BSA and incubated with cells overnight at 4°C. The following day, cells were washed with 0.01% Tween-20 in PBS, then incubated with secondary antibody for 1 hour at room temperature. After washing, cells were stained for 2 minutes with 1X DAPI to stain nuclei, then mounted onto glass slides with Vectashield (#H-1000-10, Vectorlabs). Latrunculin A-treated cells were incubated with Acti-Stain 555 Phalloidin (#PHDH1-A, Cytoskeleton) prior to DAPI staining. Cells were visualized by confocal microscopy (Zeiss LSM 710 NLO with Examiner or Zeiss LSM 880). ImageJ was used for analyses.

To calculate the percentage of nuclei containing nuclear invaginations, 100 cells per replicate were scored for the presence of invaginations or blebs. Quantification of nuclear Nesprin-1 fluorescence was performed using ImageJ. Nuclear masks were created based on DAPI staining, and mean intensities of Nesprin-1 within the mask were calculated for each replicate.

### 2.5 FRET imaging and analyses

Pre-bleach and post-bleach images were obtained using a Zeiss LSM 880 confocal microscope. After acquiring a pre-bleach image, the acceptor (Venus) was photobleached with the 515 nm laser line at 100% power with 200 iterations. A second image was acquired following photobleaching. Fluorescence intensities were obtained by selecting a region of interest around the nuclear envelope. FRET efficiency was calculated by subtracting the donor (mTFP1) intensity after photobleaching from the donor intensity prior to photobleaching, then divided by the donor after photobleaching. Each datapoint represents FRET analyzed in a single nucleus. Thirty nuclei were analyzed across three different wells (10 nuclei/well). All FRET analyses for a single experiment were performed on the same day at a single microscopy session.

## 3 Results

### 3.1 Induced expression of tau^R406W^ causes nuclear invaginations and redistribution of LINC complex proteins in cultured neuroblastoma cells

Neuronal nuclei harboring pathogenic forms of tau have significantly increased incidence of blebbing and invagination (Frost et al., 2016; Montalbano et al., 2019; Paonessa et al., 2019; Jiang and Wolozin, 2021). Studies in *Drosophila* models of tauopathy suggest that nuclear envelope invaginations and blebs are a consequence of the negative effects of pathogenic forms of tau on the actin cytoskeleton and LINC complex. In addition to nuclear pleomorphisms in human Alzheimer’s disease brain and in tau transgenic *Drosophila*, neuronal nuclei affected by tau have overall reduced levels of the B-type lamins (Frost et al., 2016), proteins that form intermediate filaments that line the internal surface of neuronal nuclei that are critical to establish nuclear structure and strength (Worman, 2012).

**FIGURE 2:**
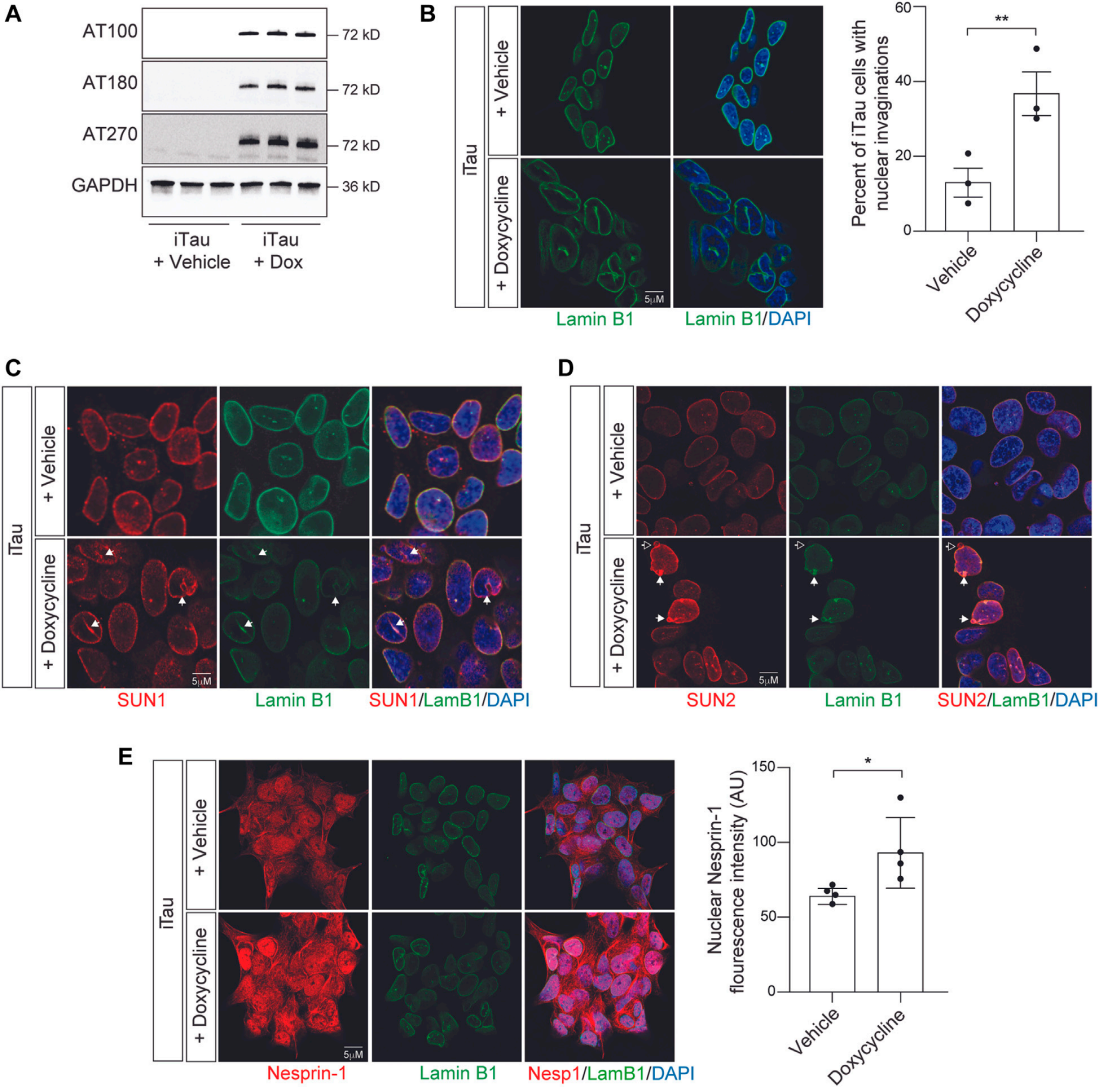
Induction of pathogenic forms of tau in iTau cells causes nuclear envelope invaginations and redistribution of LINC complex components. **(A)** Detection of disease-associated tau phosphoepitopes in iTau cells upon treatment with doxycycline based on Western blotting. **(B)** 24-h doxycycline-mediated induction of tau expression causes a significant increase in nuclear envelope invaginations compared to vehicle treated control based on lamin B1 immunofluorescence. **(C)** Relationship of SUN1 **(C)** and SUN2 **(D)** with nuclear invaginations and blebs upon doxycycline-mediated induction of tau expression in iTau cells. The empty arrow in **(D)** indicates presence of SUN2 in a nuclear bleb lacking lamin. **(E)** Doxycyline-mediated induction of tau expression in iTau cells causes a significant increase of nuclear Nesprin-1 based on immunofluorescence. *n* = 3 replicates per group, *t*-test, *p < 0.05, **p < 0.01. Error bars indicate SEM.

As a first step toward determining if the effects of pathogenic tau on nuclear structure alter nuclear tension, we developed a BE(2)-C neuroblastoma cell model that features doxycycline-inducible expression of human tau carrying the frontotemporal dementia-associated mutation *R406W* (Hutton et al., 1998) (tau^R406W^). We term this model “iTau.” We detect robust expression of human disease-associated tau phosphoepitopes in iTau cells following a 24-h induction with doxycycline compared to the vehicle treated control (Figure 2A; Supplementary Figure S1A). As a second control, we confirmed that cells with doxycycline-induced expression of GFP, “iGFP,” do not accumulate disease-associated phosphotau (Supplementary Figure S1B). As reported in human Alzheimer’s disease, tau transgenic *Drosophila* (Frost et al., 2016; Cornelison et al., 2019)*,* induced pluripotent stem cell (iPSC)-derived neurons harboring disease-associated tau (*MAPT*) mutations (Paonessa et al., 2019), and primary neurons with induced tau aggregation (Jiang and Wolozin, 2021) we find that induced expression of pathogenic tau is sufficient to drive the formation of nuclear envelope invaginations and blebs in iTau cells (Figure 2B). We do not observe an effect of doxycycline treatment alone on BE(2)-C nuclear morphology (Supplementary Figure S1C).

As previous work reports that the LINC complex causally mediates the effects of tau on nuclear architecture, and that SUN localization along the nuclear envelope is altered in tau transgenic *Drosophila* (Frost et al., 2016), we next analyzed the localization of LINC complex components SUN1, SUN2, and Nesprin-1 in iTau cells following doxycycline-induced tau expression. We observe that SUN1 lines tau-induced nuclear envelope invaginations and blebs in iTau cells (Figure 2C). SUN2 follows a similar pattern, with rare instances in which blebs are positive for SUN2 but lack lamin (Figure 2D). We detect a significant increase in nuclear localization of Nesprin-1 upon induced tau expression (Figure 2E). We next determined if induced tau expression alters overall levels of LINC complex and lamin B1 protein levels. Based on Western blotting, we do not detect significant differences in SUN1, SUN2, Nesprin-1, or lamin B1 proteins levels upon doxycycline-mediated induction of tau expression (Supplementary Figures S2A–C). These initial analyses support previous work reporting that pathogenic forms of tau alter LINC complex localization and drive formation of nuclear invaginations and blebs in other model systems and human tauopathy (Frost et al., 2016).

### 3.2 Development of a nuclear tension sensor in neuroblastoma cells

**FIGURE 3:**
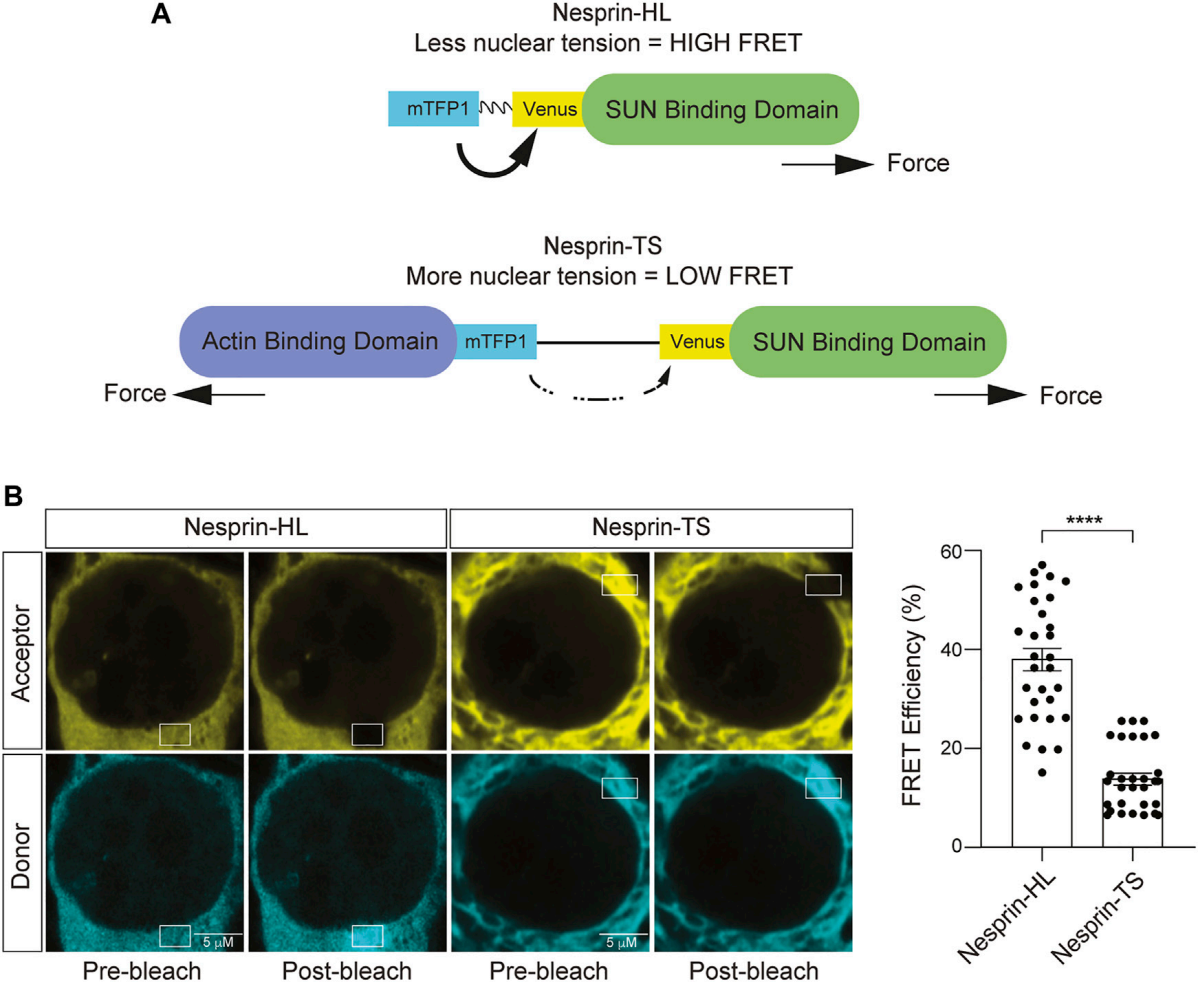
Development of a nuclear tension sensor in neuroblastoma cells. **(A)** Schematic of nesprin-HL and nesprin-TS and the inverse relationship between FRET and nuclear tension. **(B)** Acceptor photobleach-based quantification of FRET in nesprin-HL and nesprin-TS expressing BE(2)-C cells. Each datapoint represents FRET analyzed in a single nucleus. Thirty nuclei were analyzed across three different wells (10 nuclei/well), *t*-test, **p < 0.01. Error bars indicate SEM.

The LINC complex component Nesprin-2 giant (Nesprin-2G) is a large 800 kD protein that harbors two actin-binding domains at its N terminus, followed by a long stretch of spectrin-like repeats, a transmembrane domain, and a KASH domain that interacts with SUN proteins within the bilayer of the nuclear envelope (Padmakumar et al., 2004). The nesprin-TS construct consists of TSmod inserted into a miniature version of Nesprin-2G, “mini-nesprin-2G,” that lacks most of the spectrin repeats (Arsenovic and Conway, 2018). The FRET donor (mTFP1) and the FRET acceptor (Venus) are separated by an HP35 elastic peptide (Austen et al., 2015). In this system, the FRET-based nesprin-TS signal is inversely proportional to the level of tension between the actin cytoskeleton and the lamin nucleoskeleton. As a control, we utilized nesprin headless (nesprin-HL), a version of the nesprin-TS construct that lacks the actin binding domain and is thus insensitive to force (Arsenovic and Conway, 2018) (Figure 3A). 48 h after transfection of BE(2)-C cells with the nesprin-TS biosensor and the nesprin-HL control, we performed acceptor photobleach-based FRET analyses. We find significantly lower levels of FRET in nesprin-TS compared to nesprin-HL (Figure 3B), validating that the nesprin-TS biosensor functions as designed in this cell model.

### 3.3 Latrunculin A-induced depletion of filamentous actin reduces nuclear tension in neuroblastoma cells

**FIGURE 4:**
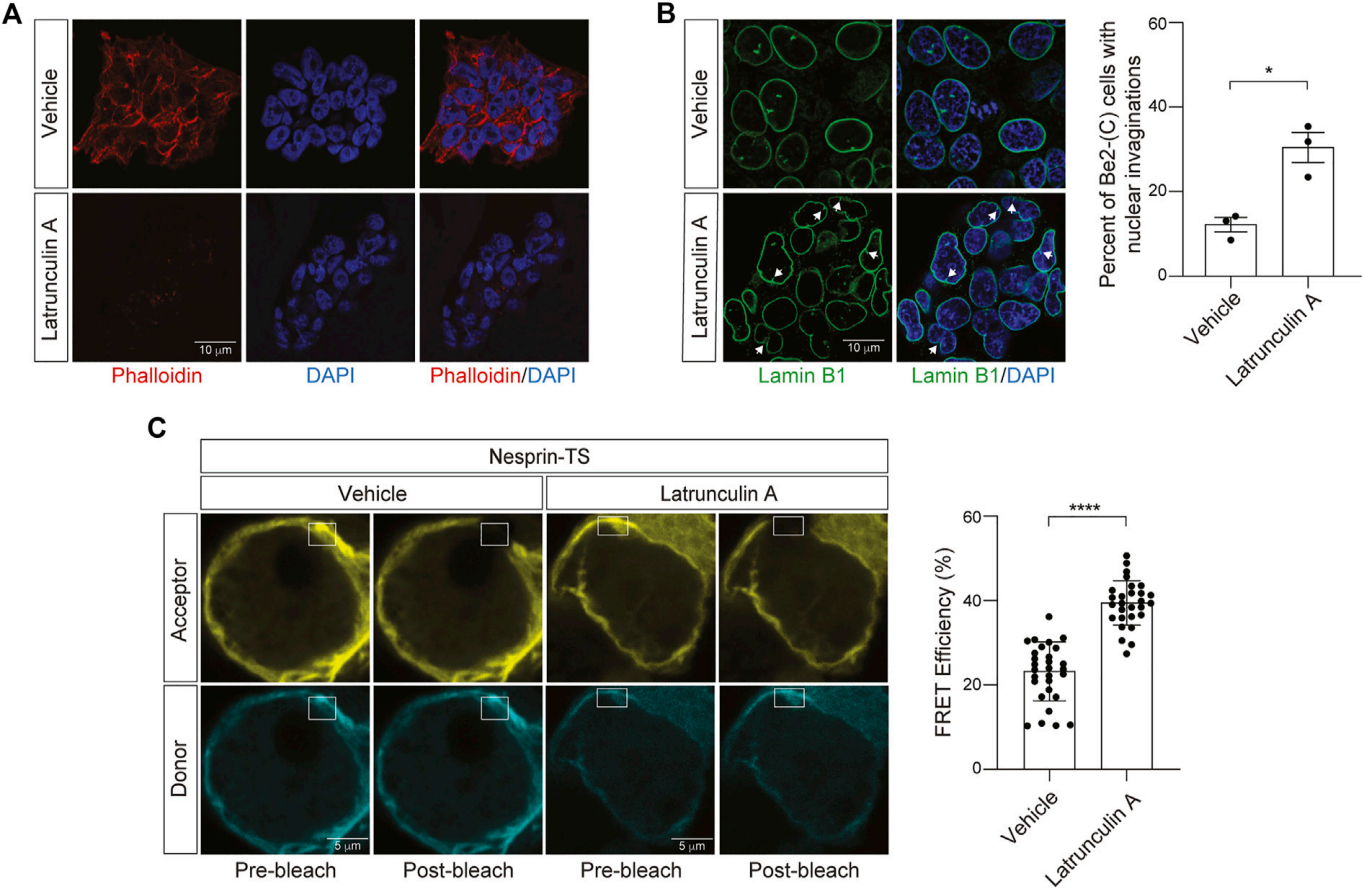
Latrunculin A-induced depletion of F-actin reduces nuclear tension in neuroblastoma cells. **(A)** Phalloidin-based visualization of F-actin in BE(2)-C cells treated with 10 µM latrunculin A or vehicle (DMSO) for 15 min. **(B)** Latrunculin A treatment drives an increase in nuclear envelope invaginations detected by lamin B1 immunofluorescence. *n* = 3 replicates per group, *t*-test, *p < 0.05. **(C)** Acceptor photobleach-based quantification of FRET in nesprin-TS expressing BE(2)-C cells upon treatment with 10 µM latrunculin A compared to vehicle. Each datapoint represents FRET analyzed in a single nucleus. Thirty nuclei were analyzed across three different wells (10 nuclei/well), *t*-test, ****p < 0.0001. Error bars indicate SEM.

Having successfully expressed nesprin-TS in BE(2)-C cells and quantified nuclear tension *via* FRET, we next asked if nesprin-TS responds to changes in nuclear tension driven by the actin cytoskeleton. Previous studies report that latrunculin A inhibits the formation of filamentous actin (F-actin) (Spector et al., 1983). We find that a 15-min treatment of neuroblastoma cells with 10 µM latrunculin A is sufficient to effectively deplete overall levels of F-actin based on phalloidin staining (Figure 4A), and that latrunculin A-mediated F-actin destabilization induces shallow invaginations of the lamin nucleoskeleton (Figure 4B). Latrunculin A treatment increases FRET efficiency in BE(2)-C cells harboring nesprin-TS (Figure 4C), indicating that F-actin destabilization is sufficient to reduce nuclear tension in cultured neuroblastoma cells.

### 3.4 Jasplakinolide-induced overstabilization of filamentous actin increases nuclear tension in neuroblastoma cells

**FIGURE 5:**
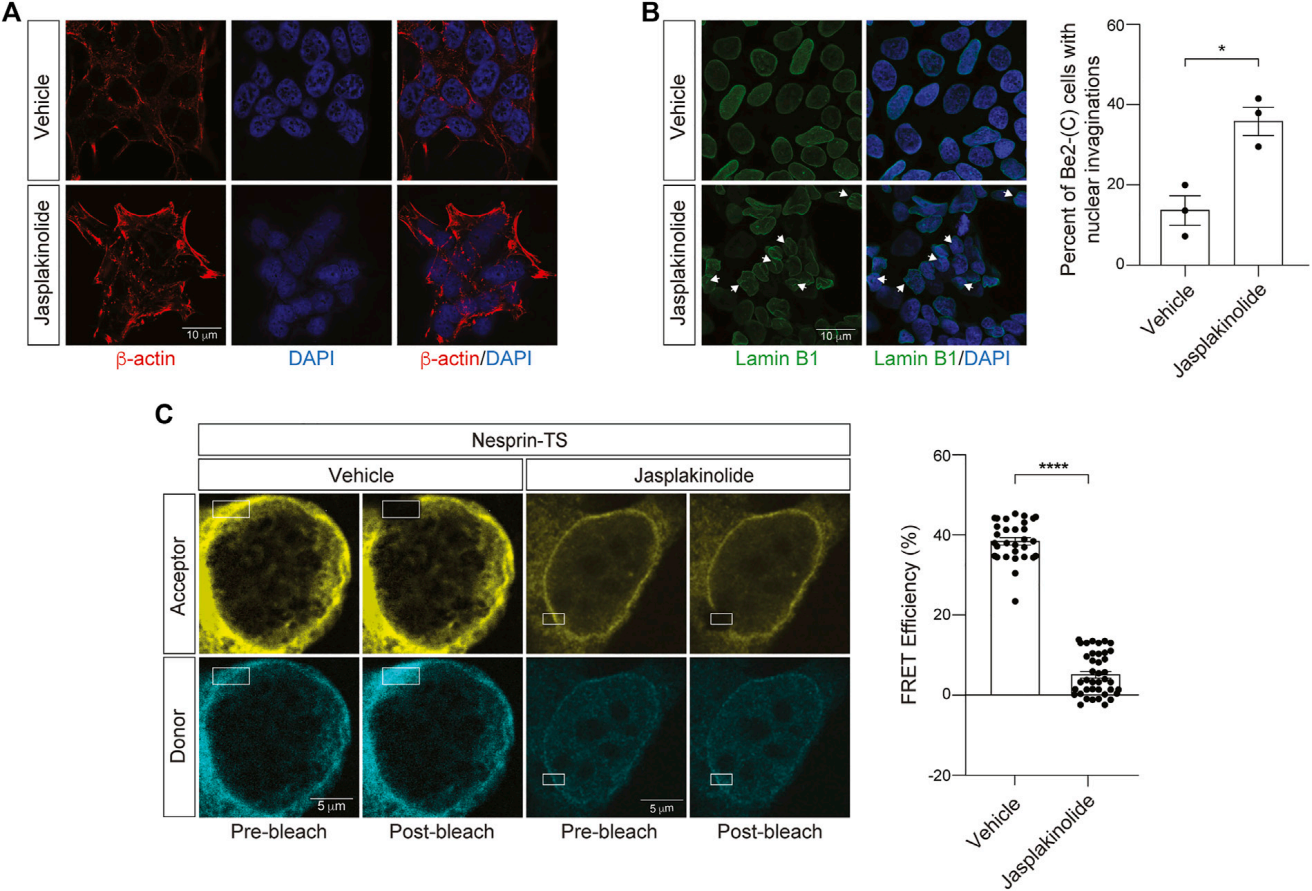
Jasplakinolide-induced elevation of F-actin increases nuclear tension in neuroblastoma cells. **(A)** Immunofluorescence-based detection of the actin cytoskeleton following a 1-h treatment of BE(2)-C cells with 0.5 µM jasplakinolide or vehicle (DMSO). **(B)** Jasplakinolide treatment induces a significant increase in nuclear envelope invaginations based on lamin B1 immunofluorescence. *n* = 3 replicates per group, *t*-test, *p < 0.05. **(C)** Acceptor photobleach-based quantification of FRET in nesprin-TS expressing BE(2)-C cells upon treatment with 0.5 µM jasplakinolide compared to vehicle. Each datapoint represents FRET analyzed in a single nucleus. Thirty nuclei were analyzed across three different wells (10 nuclei/well), *t*-test, *p < 0.05, ****p < 0.0001. Error bars indicate SEM.

We next asked if increasing cytoskeletal rigidity affects nuclear tension in BE(2)-C cells. To over-stabilize the actin cytoskeleton, we utilized jasplakinolide, a cyclo-depsipeptide that binds and stabilizes actin dimers and thus enhances nucleation of F-actin (Bubb et al., 1994). As jasplakinolide competitively inhibits the binding of phalloidin to F-actin, we visualized the actin cytoskeleton in jasplakinolide-treated cells *via* staining of cells with an antibody that detects *β*-Actin. We observe clear elevation in *β*-Actin after a 1-h treatment of BE(2)-C cells with 0.5 µM jasplakinolide (Figure 5A). Following jasplakinolide-mediated overstabilization of the actin cytoskeleton, we detect an increase in nuclear envelope invagination (Figure 5B). Using the nesprin-TS biosensor, we find that F-actin overstabilization significantly reduces FRET in BE(2)-C cells (Figure 5C), suggesting that an overly rigid cytoskeleton increases nuclear tension in this model. In some cases, the degree of nuclear tension was so high that FRET efficiency was effectively zero, indicating that the fluorophores in nesprin-TS are no longer working as a pair.

### 3.5 Expression of pathogenic tau causes a decrease in nuclear tension

**FIGURE 6:**
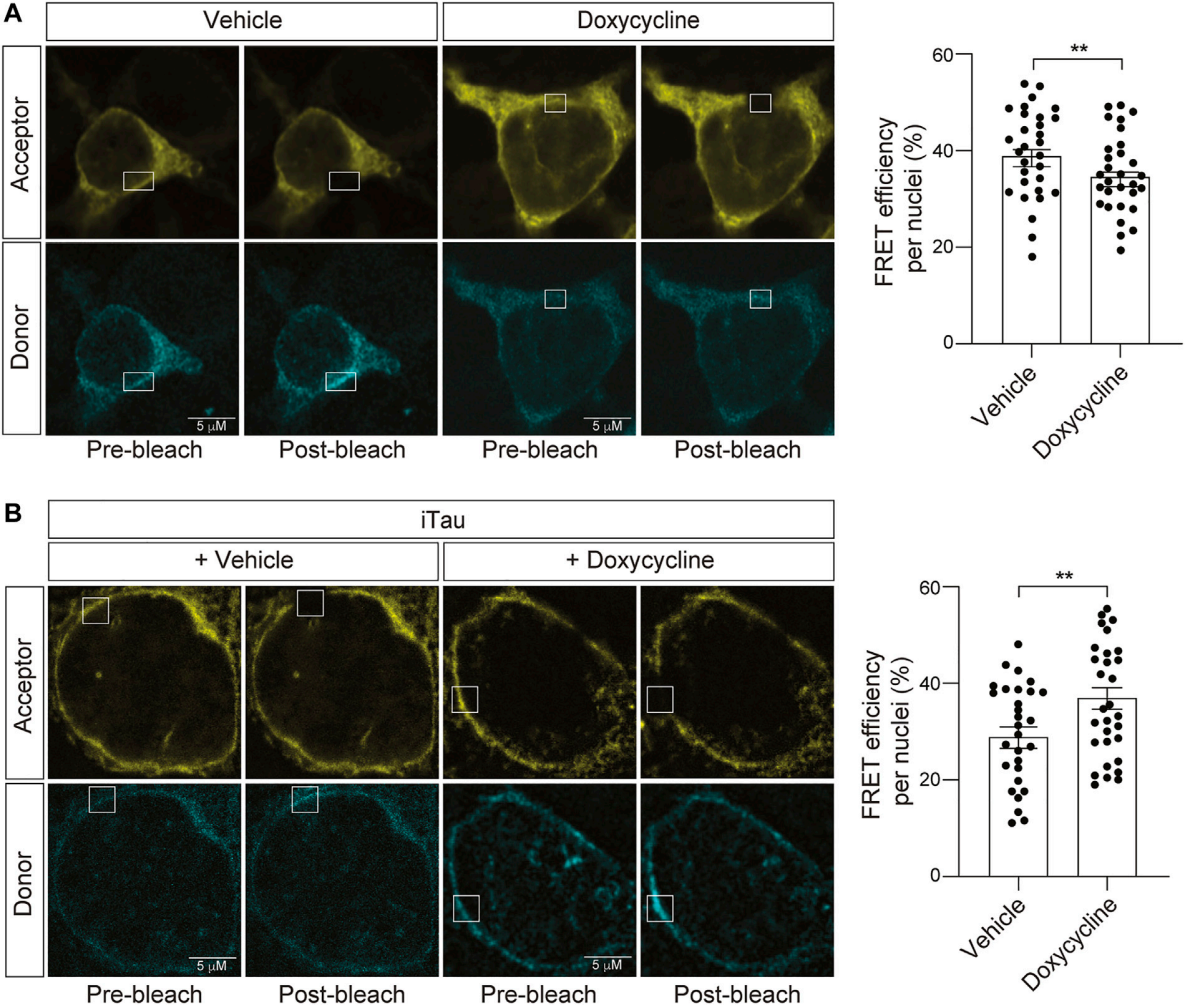
Induction of tau expression in iTau cells causes a decrease in nuclear tension. **(A)** Acceptor photobleach-based quantification of FRET in BE(2)-C cells after 24-h of doxycycline treatment reveals a doxycycline-induced increase in nuclear tension compared to vehicle (DMSO) treated cells. **(B)** Acceptor photobleach-based quantification of FRET in iTau cells after 24 h of doxycycline-mediated induction of tau expression compared to vehicle reveals a tau-induced decrease in nuclear tension. Each datapoint represents FRET analyzed in a single nucleus. Thirty nuclei were analyzed across three different wells (10 nuclei/well), *t*-test, **p < 0.01. Error bars indicate SEM.

Having established that iTau cells feature nuclear invagination and blebbing, along with relocalization of LINC complex components, and that nesprin-TS faithfully detects induced changes in nuclear tension, we next asked if nuclear tension is altered in the context of pathogenic tau. As a control for the potential effects of doxycycline alone on nuclear tension, we first determined if doxycycline treatment affects nuclear tension in the absence of induced tau expression. We find that 24 h of doxycycline treatment significantly reduces FRET, indicating that increases nuclear tension in neuroblastoma cells (Figure 6A). With this caveat in mind, we next introduced nesprin-TS into iTau cells and quantified nuclear tension *via* FRET. Despite the ability of doxycycline to reduce overall FRET in the absence of tau, we find that doxycycline-mediated induction of pathogenic tau expression significantly increases FRET, revealing a robust ability of tau to effectively decrease nuclear tension (Figure 6B).

## 4 Discussion

Pathogenic forms of tau have been linked to nuclear pleomorphism in various laboratory models and in human disease (Frost et al., 2016; Montalbano et al., 2019; Paonessa et al., 2019; Jiang and Wolozin, 2021). Several non-mutually exclusive mechanisms have been proposed to explain the effects of tau on the three-dimensional architecture of the nucleus. Studies in *Drosophila* suggest that tau-induced actin overstabilization drives relocalization of LINC complex proteins, which mediate a destabilization of the lamin nucleoskeleton, decondensation of constitutive heterochromatin, and consequent neurodegeneration (Frost et al., 2016). In line with these findings, nuclear invaginations in neurons from human Alzheimer’s disease brain tissue were found to harbor a core of filamentous actin protein. Studies in iPSC-derived neurons report that the negative effects of tau on microtubule dynamics drive nuclear envelope invagination, and that nuclear envelope invaginations contain polymerized tubulin (Paonessa et al., 2019). Both of these studies find that pathogenic forms of tau are present within nuclear envelope invaginations. A recent study in primary neurons reports that optogenetic induction of tau oligomerization deforms the nuclear envelope within minutes and causes the direct interaction of oligomeric tau species with lamin and nuclear pore proteins (Jiang and Wolozin, 2021). Based on these studies, as well as the known involvement of actin, the LINC complex, lamin, and heterochromatin as regulators of 1) nuclear stiffness, and 2) transmission and response of mechanical signals to and from the nucleus (Janota et al., 2020; Nava et al., 2020; Walker et al., 2021), we asked whether nuclei of cells affected by pathogenic tau undergo changes in nuclear tension.

Consistent with previous work in *Drosophila,* iPSC-derived neurons from patients harboring *MAPT* mutations, cultured primary cortical neurons with induced tau oligomerization, induced tau expression in HEK293 cells, and postmortem human Alzheimer’s disease brain tissue (Frost et al., 2016; Montalbano et al., 2019; Paonessa et al., 2019; Jiang and Wolozin, 2021), we observe a significant increase of nuclear invaginations and blebbing in cultured neuroblastoma cells with induced tau expression compared to controls. In addition, we find that SUN proteins closely associate with tau-induced nuclear invaginations and blebs, and that overall levels of nuclear Nesprin-1 are elevated in tau-expressing neuroblastoma cells. In line with our findings that nuclei affected by pathogenic tau feature a higher incidence of nuclear invagination and blebbing alongside relocalization of LINC complex components (Frost et al., 2016), we find that induced expression of pathological tau reduces nuclear tension.

While the studies cited above clearly indicate that cellular factors known to regulate nuclear tension are dysregulated in tauopathy, we did not focus on a particular candidate mechanism driving tau-induced reduction of nuclear tension in the current study. As pathogenic tau is reported to cause overstabilization of the actin cytoskeleton (Fulga et al., 2007; He et al., 2009; Cabrales Fontela et al., 2017), we thought perhaps that pathogenic tau would *increase* stiffness of nuclei based on our finding that jasplakinolide-induced F-actin enrichment was sufficient to increase nuclear tension. On the other hand, lamin B1 reduction and nuclear softening occur in cell culture-based models of lung and breast cancer (Jia et al., 2019; Fischer et al., 2020), and knockout of *lamin A/C* or induction of heterochromatin decondensation are both sufficient to decrease nuclear stiffness (Lammerding et al., 2004; Lammerding et al., 2006; Nava et al., 2020). While lamin B1 deficiency is reported to drive nuclear blebbing in the absence of changes to nuclear stiffness in mouse embryonic fibroblasts (Lammerding et al., 2006), overexpression of lamin B1 increases nuclear stiffness in HEK293 and N2a cells, as well as in cells from patients with a genetic duplication of the gene encoding lamin B1 (Ferrera et al., 2014). Based on these studies, we speculate that the reduction in nuclear tension that we observe in neuroblastoma cells with induced tau expression is a consequence of a destabilized nucleoskeleton (Frost et al., 2016; Montalbano et al., 2019) and decondensation of constitutive heterochromatin (Frost et al., 2014).

Due to its direct interactions with the actin cytoskeleton and the lamin nucleoskeleton, the LINC complex detects and transmits mechanical information to and from the nucleus (Janota et al., 2020). Increased nuclear tension is reported to induce the recruitment of lamin A/C to the LINC complex (Guilluy et al., 2014), suggesting that mechanical pull on nesprins may trigger a reinforcement of the lamin nucleoskeleton. Studies in skin epidermis progenitor cells provide insight into the biological benefit of nuclear tension changes that result from external force (Nava et al., 2020). In these studies, nuclear softening in response to mechanical force was found to protect cells against the accumulation of DNA damage that occurs in response to cellular stretching. While we do not currently know if tau-induced nuclear softening causally mediates neuronal death, it is possible that reduced nuclear tension could serve as a compensatory response to protect DNA from mechanical force-induced damage in tauopathy. While our data suggest that induced expression of disease-associated *MAPT* mutant tau causes loss of nuclear tension, it is important to note that we do not currently know if physiological forms of tau also regulate the mechanical properties of the nucleus in non-disease settings.

In addition to its role as a mechanosensor (Kirby and Lammerding, 2018), the nucleus serves as a mechanoresponsive organelle that facilitates the cellular response to an altered mechanical environment. Nuclear adaptations to mechanical stress involve a range of biological processes including differential expression of “mechanosensitive genes,” posttranslational protein modifications and localization, changes in nuclear pore permeability and mechanosensitive ion channels, and cellular lineage determination (Maurer and Lammerding, 2019). Increased “leakiness” of nuclear pores (Eftekharzadeh et al., 2018) and elevation of RNA export in models of tauopathy (Cornelison et al., 2019) are in line with the effects of altered nuclear mechanotransduction on nuclear pores. Similarly, we have previously reported that neuronal nuclei of iPSC-derived neurons from human Alzheimer’s disease cases as well as tau transgenic *Drosophila* feature depletion of nuclear calcium (Mahoney et al., 2020). While these cellular phenotypes are consistent with the potential effects of pathogenic forms of tau on nuclear mechanotransduction, it remains to be determined whether tau-induced changes in nuclear stiffness are a direct driver of nuclear calcium changes and nuclear pore permeability in tauopathy.

While our study is the first to investigate nuclear tension in neurons and provides the first insights into the effects of pathogenic tau on nuclear mechanics, we note several limitations of our approach. As the human brain is a rather soft tissue with a Young’s modulus ranging from ∼1.389–1.895 kPa (Budday et al., 2015), neurons grown on glass coverslips experience a significantly different environment than neurons within the human brain. In addition, the constant mechanical stress generated by brain vasculature is not present in our system, nor are the diverse cell types that make up a living brain. As various cancers are associated with changes in nuclear tension, our use of an immortalized cell line may contribute to the effects of tau on nuclear tension. Finally, while we speculate that our findings are not restricted to the *MAPT R406W* mutation, as sporadic human tauopathy and various models of tauopathy featuring wild-type forms of tau also exhibit nuclear pleomorphism, future studies are required to determine if toxic forms of wild-type tau also induce nuclear softening.

The current understanding of nuclear mechanotransduction is almost completely based on non-neuronal cell types. Our findings provide proof-of-principle data that provide rationale for future study of the effects of tau on nuclear mechanotransduction and the development of systems to better study nuclear mechanics of neurons in more physiological environments such as iPSC-derived cerebral organoids or *in vivo* models. With the knowledge that cells of the brain are indeed subject to mechanical stress, our analyses provide the first insights into the response of neuronal nuclei to external force from the actin cytoskeleton, as well as changes to nuclear tension that occur as a consequence of pathogenic tau.

## Data Availability

The original contributions presented in the study are included in the article/Supplementary Material, further inquiries can be directed to the corresponding author.
