# Supplementary material for: Pathogenic tau decreases nuclear tension in cultured neurons
Source: Front Aging. 2023 Jan 23;4:1058968. doi: 10.3389/fragi.2023.1058968 (PMC9901542; doi:10.3389/fragi.2023.1058968)
Supplement: Supplementary file 1 [file DataSheet1.PDF]

Supplementary Material

Pathogenic tau decreases nuclear tension in cultured neurons

Claira Sohn[1], Jiacheng Ma, PhD[2], William J Ray, PhD[2], Bess Frost, PhD[1]

*** Correspondence:** Bess Frost, PhD bfrost@uthscsa.edu

1 Supplementary Figures and Tables

1.1 Supplementary Figures

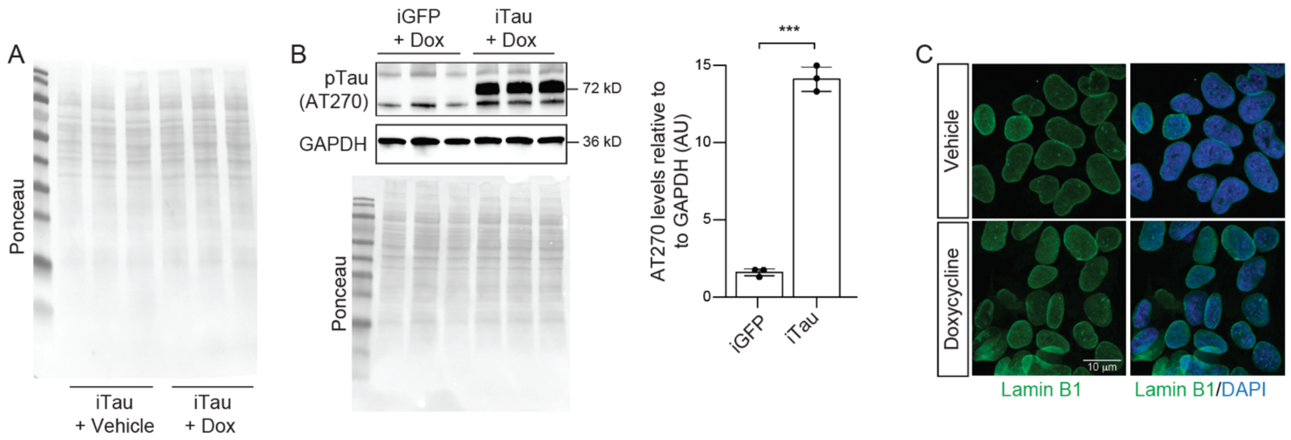

Supplementary Figure 1. Analyses of BE(2)-C cells after 24-hour doxycycline exposure. A. Ponceau-based detection of protein levels of Western blot presented in Fig. 2A. **B.** pTau (AT270) levels in iTau versus iGFP cells following 24 hours of doxycycline-mediated transgene induction. **C.** Immunofluorescence-based visualization of the lamin nucleoskeleton indicates that doxycycline treatment does not alter nuclear architecture in BE(2)-C cells. n=3 replicates per group, t-test, ***p<0.001. Error bars indicate SEM.

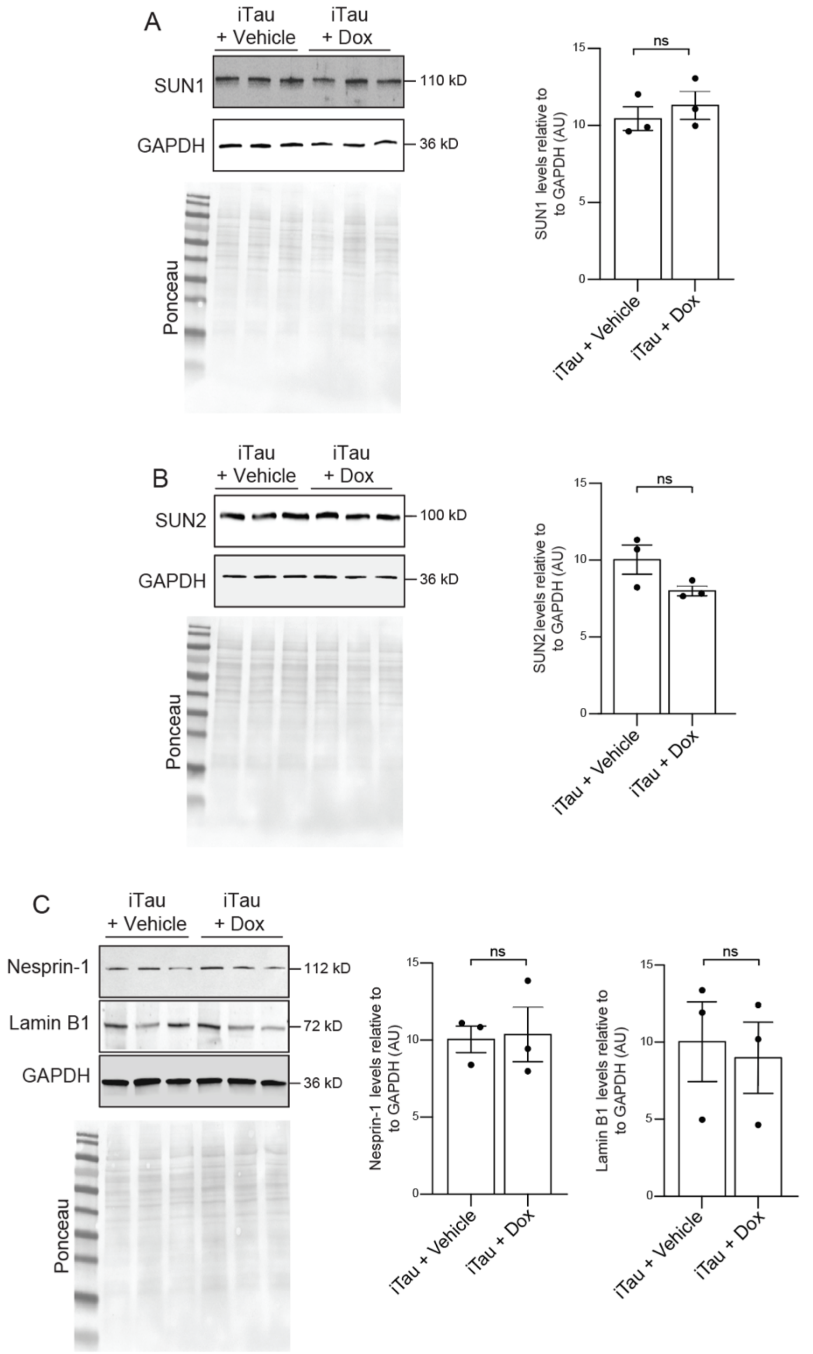

Supplementary Figure 2. Induction of tau expression does not change LINC or lamin B1 protein levels in iTau cells. Protein levels of SUN1 (A), SUN2 (B), Nesprin-1 and lamin B1 (C) in iTau cells after 24 hours of doxycycline-induced tau expression. n=3 replicates per group, t-test, ns = not significant. Error bars indicate SEM.
